# Body Composition, Microbiome and Physical Activity in Workers Under Intermittent Hypobaric Hypoxia

**DOI:** 10.3390/nu17243919

**Published:** 2025-12-15

**Authors:** Jorge Torres-Mejías, Karem Arriaza, Francisco Mena, Evangelina Rivarola, Patricio Paredes, Husam Ahmad, Iván López, Daniel Soza, José Luis Pino-Villalón, Miguel Ángel López-Espinoza, Samuel Duran-Agüero, Eugenio Merellano-Navarro

**Affiliations:** 1 Doctoral Program in Physical Activity Sciences, Faculty of Education Sciences, Universidad Católica del Maule, Talca 3530000, Chile; jorge.torres.02@alumnos.ucm.cl; 2 High Altitude Medicine Research Center (CEIMA), Arturo Prat University, Iquique 1100000, Chile; 3 Centro de Investigación Andino para la Altitud Geográfica, CIAAG, Santiago 7500000, Chile; fmena@ciaag.cl (F.M.); evangelina.rivarola@unc.edu.ar (E.R.); hahmad@ciaag.cl (H.A.); 4 Escuela de Nutrición, Facultad de Ciencias Médicas, Universidad Nacional de Córdoba, Córdoba PC 5000, Argentina; 5 Atacama Large Millimeter/Submillimeter Array, ALMA Observatory, Santiago 7630355, Chile; ivan.lopez@alma.cl (I.L.);; 6 Escuela de Nutrición y Dietética, Facultad de Salud, Universidad Santo Tomás, Santiago 8370003, Chile; 7 Carrera de Nutrición y Dietética, Facultad de Ciencias de la Salud, Universidad Adventista de Chile, Chillán 3780000, Chile; miguellopez@unach.cl; 8 Escuela de Nutrición y Dietética, Facultad de Ciencias de la Rehabilitación y Calidad de Vida, Sede Los Leones, Universidad San Sebastián, Santiago 7500000, Chile; samuel.duran@uss.cl; 9 Department of Physical Activity Sciences, Faculty of Education Sciences, Universidad Católica del Maule, Talca 3530000, Chile

**Keywords:** body composition, microbiome, physical activity, intermittent hypobaric hypoxia, high altitude, ALMA observatory

## Abstract

**Background/Objectives:** Intermittent hypobaric hypoxia (IHH) induces various physiological and metabolic adaptations. This study aimed to investigate the effects of a seven-day IHH exposure on nutritional status, body composition, gut microbiota, movement intensity, and energy expenditure in 10 workers. **Methods**: A pre–post comparative design was employed, with measurements taken at the beginning and end of the exposure period. Nutritional status, body composition, and phase angle (PhA) were assessed via bioelectrical impedance analysis (BIA). Gut microbiota composition was analyzed through fecal DNA extraction and qPCR for specific bacterial families. Movement intensity and energy expenditure were monitored using accelerometry. An initial statistical analysis was performed, which included paired *t*-tests and Wilcoxon signed-rank tests. **Results:** A significant increase in PhA (mean difference: 0.40; *p* = 0.0053 for *t*-test, *p* = 0.0136 for Wilcoxon) and a significant decrease in BMI (mean difference: −0.38; *p* = 0.0311 for *t*-test, *p* = 0.0546 for Wilcoxon). **Conclusions**: While the original paper reported no significant changes in nutritional status or body composition, our re-analysis suggests a significant change in BMI. The original paper also reported significant changes in specific gut bacterial families (butyrate-producing bacteria, *p* = 0.037; Lactobacillus species, *p* = 0.006). Physical activity levels remained consistently low.

## 1. Introduction

Approximately 500 million people live at altitudes above 1500 m above sea level (m), a zone known as “altitude,” while high altitude (HA) is defined as elevations above 3000 m and extreme altitude (EA) as those above 5000 m [1]. Individuals at these altitudes live under conditions of hypobaric hypoxia (HH) [2], which triggers inter- and intracellular signals to regulate oxygen utilization [3], leading to adaptive mechanisms to counteract the reduction in partial pressure of oxygen (PpO2) [4,5], thereby reducing gas exchange and affecting maximal oxygen consumption (VO2max) [6].

Not only do native populations of the Altiplano experience acclimatization to hypobaric hypoxia (HH), so do visitors and workers who ascend to HH areas [7], such as mining personnel and astronomical observatories like the Atacama Large Millimeter/Submillimeter Array (ALMA) [8]. In Chile, this activity is mainly carried out in areas with a permanent state of HH and low ambient humidity [9,10], conditions that increase heart rate and inspiratory volume, while reducing VO2max, which is associated with an increase in C-reactive protein (CRP) [11]. These workers in HH conditions are referred to as “intermittent hypobaric hypoxia induces altitude acclimation” (IHH) [12].

Another notable effect associated with exposure to HA is the alteration of the intestinal microbiota (IM) [13]. A predominance of the firmicutes phylum has been documented, which can reach up to 62% of the bacterial community [14]. Studies on acute exposure to HA have also reported various changes, including a reduction in the abundance of bifidobacterium [15] and Lactobacillus [16], although there are contradictory findings in the literature [17]. These alterations are noteworthy given the beneficial role of these bacterial genera (probiotics) in human health [18,19]. Changes in the composition of the microbiota induce variations in its production of metabolites, particularly in short-chain fatty acids (SCFAs) [20]. Among these, butyric acid is notable for its systemic physiological effects [21]. Notably, strains of Faecalibacterium spp., major producers of butyrate, are associated with a more favorable intestinal microbial profile [22]. It has been suggested that dietary fiber supplementation could attenuate the effects of HH, partly by positively modulating IM [23].

In HA environments, physiological responses include activation of the sympathetic nervous system, pulmonary vasoconstriction, increased pulmonary artery pressure, and elevated heart rate [24,25]. At the cognitive level, a decrease in concentration may occur, with one of the most serious disorders being the appearance of acute mountain sickness (AMS), which progresses to severe symptoms such as pulmonary and cerebral edema [26]. Although oxygen supplementation is used in ALMA, our study does not evaluate its direct impact on physiological markers and changes in microbiota. However, this oxygen enrichment is a simple and effective strategy to improve sleep quality, cognitive performance, and work efficiency under HH conditions [27,28].

While prolonged hypoxic exposure can pose serious health risks [29], HH also induces beneficial adaptations. For example, Mallet RT et al. (2021) [30] demonstrated that hypoxia can enhance myocardial resistance to ischemia and improve perfusion by increasing β-adrenergic activity, promoting moderate reactive oxygen species (ROS) formation, and activating genetic factors such as Nrf2 and HIF-1. These changes lead to greater ATP production via anaerobic pathways, improved Ca^2+^ transport across cell membranes, suppression of necrosis, and preservation of mitochondrial integrity—mechanisms that may confer cardio protection.

In addition to these cellular adaptations, HH has been linked to increased energy expenditure during physical work and improvements in glucose and lipid metabolism, which could theoretically reduce body Fat Mass percentage (%FM) in individuals exposed to such environments [31,32]. These changes can be assessed through nutritional and body composition evaluations using multifrequency bioelectrical impedance analysis (BIA) [33], which also enables calculation of the phase angle (PhA)—a robust marker of cell membrane integrity and a reliable clinical tool [34].

Therefore, the present study aims to identify potential changes in nutritional status, body composition, gut microbiome, movement intensity, and energy expenditure among individuals working under intermittent conditions during a seven-day work shift at the ALMA Observatory.

## 2. Materials and Methods

This preliminary single-group pilot study was conducted over seven days, with pre- and post-shift assessments, in 10 ALMA Observatory workers (maintenance, science, and engineering personnel) located at 2950 m. The staff follow a 7 × 7 shift system, alternating seven days at sea level with seven days of intermittent exposure to hypobaric hypoxia. This sample size was sufficient to detect an effect size of at least 1.00, with a two-tailed α = 0.05 and 1 − β = 0.80. Participants were recruited through non-probability convenience sampling. Workers were invited to participate on the first day of their shift upon entering the Observatory facilities. Ten workers agreed to participate; all were men, aged 30 to 52, and reported no medical conditions. In accordance with Chilean law—Decreto Supremo Nº 594—all had previously passed the mandatory pre-employment medical examination required for work at high altitude under intermittent hypobaric conditions.

Inclusion criteria were as follows: (a) being employed by the observatory; (b) having more than one year of work experience at the site, and (c) not having a diagnosed non-communicable disease. Exclusion criteria were as follows: (a) having heart disease or a pacemaker and (b) residing in geographic areas above 1600 m.

Assessments were performed at the beginning of the shift (between 7:00 and 8:00 AM) and again on the last day of the shift at the same time. All assessments were conducted during the Southern Hemisphere spring (November), under a partial barometric pressure of 530 ± 2 mmHg at an altitude of 2950 m. The participants’ diets were not individually planned; instead, they followed the meal options provided by the catering company, which included fish, lean meats, low-calorie choices, and a free selection of desserts. However, observatory staff were free to choose the combinations and portions they preferred; therefore, it was not possible to accurately determine the subjects’ dietary intake in this study.

### 2.1. Nutritional Status and Body Composition Assessment

Before starting the work shift and after an overnight fast of at least eight hours, weight and height were measured using a Seca model 769 scale (Hamburgo, Germany), with a built-in stadiometer (1 mm graduation). Measurements were taken with minimal clothing. Waist circumference was then measured, located between the last rib and the iliac crest. Finally, hip circumference was measured at the most prominent area of the buttocks. All measurements were taken three times, and the average of the three was used. A Seca model 700 fixed measuring tape was used, and the measurements were performed by a nutritionist specializing in body composition analysis with ISAK Level 2 training [35].

Fixed measuring tape was used, and the measurements were performed by a nutritionist specializing in body composition analysis with ISAK Level 2 training [35].

Body composition was assessed with participants lying supine on a clinical table, arms at their sides and legs extended. Following the Bodystat Quadscan 4000 (Chester House, Main Road, Sulby, Isle of Man, IM7 2HJ, British Isles) bioelectrical impedance protocol, two electrodes were placed on the right hand and foot. The device provided measurements of total body water percentage (%WCT), %FM, fat-free mass percentage (%FFM), and phase angle (PhA), calculated from the resistance-reactance ratio obtained at frequencies of 5, 50, 100, and 200 kHz [36].

### 2.2. Gut Microbiota Analysis

Each participant provided two stool samples, one on the first day and one on the last day of the work shift, collected in sealed, sterile containers. Samples were processed in duplicate immediately at the ALMA Observatory laboratory (2950 m). DNA was extracted from 200 mg of stool using the QIAamp DNA Stool Mini Kit (QIAGEN, Hilden, Germany) and quantified at the Center for Integrative Ecology, University of Talca, using quantitative PCR (qPCR).

Populations of *firmicutes*, *butyrate-producing bacteria*, *lactobacillus* spp. and *bifidobacteria* spp. were quantified with the following primers: Firm934F (5′-GGAGYATGTGGTTTAATTCGAAGCA-3′), Firm1060R, (5′ AGCTGACGACAACCATGCAC 3′), BcoA Tscr-F (5′ GCIGAICATTTCACITGGAA-YWSITGGCAYATG 3′) and BcoA Tscr-R (5′ CCTGCCTTTGACATRTCIACRAANGC 3′), Lacto-F (5′ AGCAGTAGGGAATCTTCCA 3′) and Lacto-R (5′ CACCGCTACACATGGAG 3′), BifF (5′ CACCCGTTTCCAGGAGCTATT 3′) and BifR (5′ GCGTGCTTAACACATGCAAGTC 3′), respectively.

Amplification was performed on the Mic qPCR platform (BMS, Mulgrave, VIC, Australia) using the Brilliant II SYBR^®^ Green QPCR Master Mix kit (Stratagene, La Jolla, CA, USA). Each reaction contained 12.5 µL of SYBR mix, 0.75 µL of forward primer, 0.75 µL of reverse primer, 8.5 µL of nuclease-free water, and 2 µL of DNA. The cycling conditions were initial denaturation at 95 °C for 10 min; 45 cycles at 96 °C for 30 s, annealing at 55 °C for 60 s, and extension at 72 °C for 60 s for *Bifidobacterium* spp. The annealing temperatures were 56 °C for *Lactobacillus*, 53 °C for *butyrate-producing bacteria*, and 60 °C for *Firmicutes*. Microbial abundance was quantified from Cq values obtained by qPCR assays targeting the 16S rRNA Cq values [37].

### 2.3. Determination of Physical Activity Level

Participants wore ActiGraph wGT3X-BT accelerometers (ActiGraph, Pensacola, FL, USA) on the right hip throughout the work shift (seven consecutive days). Data were collected at 100 Hz and downloaded using ActiLife 6.13.4 software in 10 s epochs. Sedentary behavior and physical activity intensities were classified according to the cutoff points proposed by Arias-Palencia et al. [38].

### 2.4. Ethical Aspects

This study was approved by the Ethics Committee of the University of Santo Tomás, South Zone (Resolution ID 129-17). Participants signed informed consent. All procedures followed the Declaration of Helsinki for Scientific Work Involving Human Subjects [39].

### 2.5. Statistical Analysis

Body composition measurements were compared using the Student *t* test for paired samples; for gut microbiota, the nonparametric Wilcoxon test was used. Linear regression analyses were performed to examine associations between phase angle and changes in body composition over the 7-day work shift. Coefficients of determination were calculated, and statistical significance was set at *p* < 0.05. Analyses were performed using R-Studio V 4.4.2.

## 3. Results

**Figure 1:**
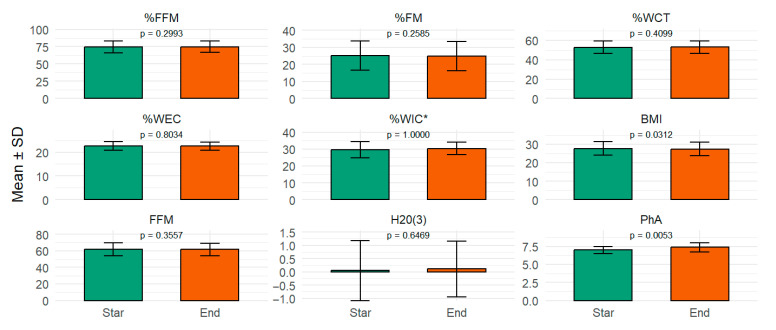
Nutritional status and body composition in three compartments: Phase Angle, fat mass, fat-free mass, and water mass. * Wilcoxon test. All other comparisons were performed using Student’s *t*-test. Percentage and values of change at the start of the work shift (green) and at the end of the work shift (orange). PhA: Phase angle values. BMI: Body mass index. FFM: Fat-free mass. H_2_O(3): Third space water. %WEC: Extracellular water percentage. %WIC: Intracellular water percentage. %WCT: Total body water percentage. %FM: Fat mass percentage. %FFM: Fat-free mass percentage.

Figure 1 shows the subjects’ body composition values on the first and last day of the work shift, under HH conditions at 2950 m. A significant change was observed in the subjects’ phase angle (PhA) and body mass index (BMI). The PhA increased from 6.98 to 7.38, with a mean difference of 0.40 (95% CI: 0.15–0.65; *p* = 0.0053), indicating a change in the integrity and function of cell membranes under exposure to relative humidity (HH).

**Figure 2:**
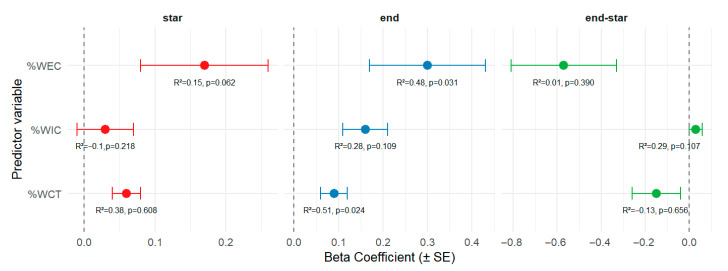
Relationship between phase angle and body water changes at the beginning and end of the shift, adjusted for weight. %WEC = Extracellular Water percentage; %WIC = Intracellular Water percentage. %WCT = Total Body Water percentage.

Figure 2 shows a significant relationship between PhA and the percentage of body water corrected for body weight, both intracellular and extracellular. Intracellular water percentage (%WIC) showed the strongest association with PhA, suggesting that intracellular hydration played a more significant role than extracellular hydration.

**Figure 3:**
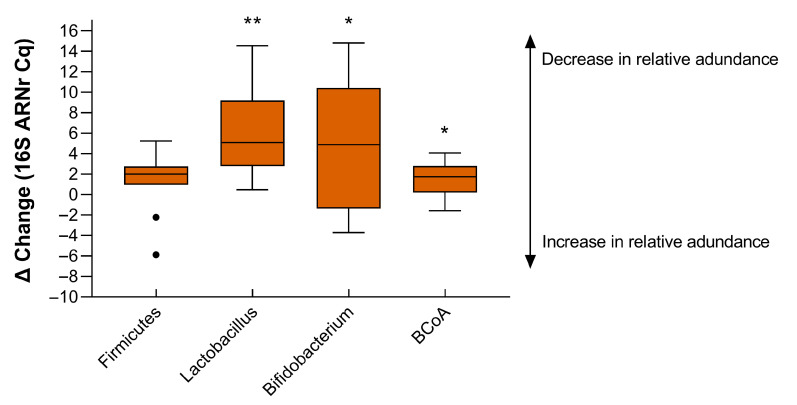
Gut microbiota: Changes in gut microbiota under hypobaric hypoxia conditions (N = 10). * *p* < 0.05, ** *p* < 0.01, BCoA: *Butyrate-producing bacteria*. Values indicate the mean and standard deviation of the fluorescence detection value (Ct). Wilcoxon rank test.

Figure 3 illustrates a significant change in specific gut bacterial families, particularly *butyrate-producing bacteria* (BCoA) (*p* = 0.037), *Lactobacillus species* (*p* = 0.006) and *Bifidobacterium*. These findings highlight the sensitivity of the gut microbiome to IHH. Physical activity levels were consistently low, with sedentary behavior averaging 6246 min over the 7 days of measurement, indicating a predominantly sedentary work environment.

## 4. Discussion

One of the most notable and significant findings was the increase in PhA in all participants, suggesting a change in cell membrane integrity during HH exposure [34]. This change was likely driven by water exchange between intracellular and extracellular compartments, given that electrical current flows through aqueous media [2,40]. Interestingly, when the relationship between PhA and body water was corrected for body weight, %WIC showed a stronger correlation than %WEC, contrary to previous reports, since higher extracellular water concentrations are typically associated with inflammatory responses [41]. This finding might reflect chronic acclimation to HH in this population [2,9]. Furthermore, this water exchange is likely influenced by metabolic stress mediated by proinflammatory cytokines under HH conditions [40], which can enhance glucose and lipid metabolism; however, this was not assessed in our study [31,32,42]. Therefore, in subjects working under HH conditions, an increase in energy expenditure and a decrease in %FM could be observed, which could modify BMI and nutritional status [31,32,43,44]. However, this was not observed in our study, possibly due to the short duration of exposure and the sedentary nature of the participants’ activities.

The study of these four bacterial phyla in this population of 10 healthy adult male professionals in the fields of mechanical maintenance, science, and engineering is motivated by the fact that they are the most frequently reported bacterial families in the literature [14], and the observed reduction in the *Lactobacillus*, *Bifidobacterium*, and *BCoA bacterial* families could affect immune system function [44]. Similarly, the change in *BCoA bacteria* could generate intestinal inflammation [13,45]. Although these microbiota changes could be partly explained by the specific diet provided during the work shift, which is planned by nutritionists, the nutritional strategy does not account for the physiological impact of HH [8]. These shifts could have implications for gut health and overall metabolic function, as these bacteria play crucial roles in maintaining gut barrier integrity and producing beneficial short-chain fatty acids [18,19,20,21]. The consistently low physical activity levels among workers at ALMA Observatory underscore the importance of considering lifestyle factors in conjunction with environmental stressors like IHH. Future studies could explore the interplay between physical activity, gut microbiota, and body composition changes under IHH.

Living under HH conditions induces physiological, morphological, and cellular adaptations [6,43], yet the precise cellular membrane factors involved remain unclear. Although our sample size was small, this is the first study to examine intestinal microbiota changes under HH conditions in Chile. Given that a substantial portion of the national workforce operates under such environmental conditions, it is essential to continue conducting comprehensive assessments—including nutritional, physical activity, and microbiota evaluations—in larger cohorts to establish evidence-based guidelines that safeguard worker health.

Limitations and Future Directions: The primary limitation of this study remains its small sample size (N = 10), which restricts the generalizability of findings and the power to detect subtle effects. The enhanced statistical recommendations, such as LMMs and multivariate microbiome analyses, would allow for a more nuanced understanding of the data, even with a small sample, by accounting for within-subject variability and the complex nature of microbiome data. Future research should aim for larger sample sizes, incorporate a control group, and include a broader range of physiological and biochemical markers (e.g., inflammatory markers, metabolic hormones) to provide a more holistic picture of adaptation to IHH. Additionally, a more detailed analysis of physical activity patterns and their correlation with physiological and microbiological changes would be beneficial.

## 5. Conclusions

This study demonstrates that seven days of exposure to intermittent hypobaric hypoxia induces significant alterations in phase angle and, as revealed, a significant decrease in BMI. Furthermore, changes were observed in specific gut microbiota families, while physical activity levels remained low. These findings highlight the human body’s adaptive responses to HHI, particularly about cellular health and gut microbiome composition. Finally, we believe that studying these physiological and nutritional conditions in people working in hypobaric environments is crucial given that in Chile, there are over 700,000 people in various sectors exposed to high-altitude conditions, a number that is constantly increasing, whether in mining, astronomical observation, or other fields. Therefore, it is necessary to understand these metabolic variables to improve the quality of life of this population, considering nutritional supplementation for work at altitude or foods that can prevent potential changes in the gut microbiota. Along with this, we believe it is necessary to continue conducting studies on this population, as we need to understand the behavior of the human body once these individuals finish working and begin their retirement period.

## Data Availability

The data are not publicly available due to privacy or ethical restrictions, and privacy of the workers.

## References

[1] Tremblay J.C., Ainslie P.N. (2021). Global and country-level estimates of human population at high altitude. Proc. Natl. Acad. Sci. USA.

[2] Getu A. (2022). Ethiopian Native Highlander’s Adaptation to Chronic High-Altitude Hypoxia. Biomed. Res. Int..

[3] O’Brien K.A., Murray A.J., Simonson T.S. (2022). Notch Signaling and Cross-Talk in Hypoxia: A Candidate Pathway for High-Altitude Adaptation. Life.

[4] Berger M.M., Luks A.M. (2023). High Altitude. Semin. Respir. Crit. Care Med..

[5] Apte C.V. (2023). Barometric Pressure at High Altitude: Revisiting West’s Prediction Equation, and More. High. Alt. Med. Biol..

[6] Wehrlin J.P., Hallén J. (2006). Linear decrease in $\dot{V}$O_2max_ and performance with increasing altitude in endurance athletes. Eur. J. Appl. Physiol..

[7] Lang M., Bilo G., Caravita S., Parati G. (2021). Blood pressure and altitude: Physiological responses and clinical management [Blood pressure and high altitude: Physiological response and clinical management]. Medwave.

[8] Atacama Large Millimeter/Submillimeter Array (ALMA) (ESO/NAOJ)/NRAO). About ALMA. https://www.almaobservatory.org/es/inicio/.

[9] Pizarro-Montaner C., Cancino-Lopez J., Reyes-Ponce A., Flores-Opazo M. (2020). Interplay between rotational work shift and high altitude-related chronic intermittent hypobaric hypoxia on cardiovascular health and sleep quality in Chilean miners. Ergonomics.

[10] Calderon-Jofre R., Moraga D., Moraga F.A. (2022). The Effect of Chronic Intermittent Hypobaric Hypoxia on Sleep Quality and Melatonin Serum Levels in Chilean Miners. Front. Physiol..

[11] Pedreros-Lobos A., Calderón-Jofré R., Moraga D., Moraga F.A. (2021). Cardiovascular Risk Is Increased in Miner’s Chronic Intermittent Hypobaric Hypoxia Exposure from 0 to 2500 m?. Front. Physiol..

[12] Viscor G., Torrella J.R., Corral L., Ricart A., Javierre C., Pages T., Ventura J.L. (2018). Physiological and Biological Responses to Short-Term Intermittent Hypobaric Hypoxia Exposure: From Sports and Mountain Medicine to New Biomedical Applications. Front. Physiol..

[13] Liu D., Chen D., Xiao J., Wang W., Zhang L.-J., Peng H., Han C., Yao H. (2024). High-altitude-induced alterations in intestinal microbiota. Front. Microbiol..

[14] Das B., Ghosh T.S., Kedia S., Rampal R., Saxena S., Bag S., Mitra R., Dayal M., Mehta O., Surendranath A. (2018). Analysis of the Gut Microbiome of Rural and Urban Healthy Indians Living in Sea Level and High Altitude Areas. Sci. Rep..

[15] Kleessen B., Schroedl W., Stueck M., Richter A., Rieck O., Krueger M. (2005). Microbial and immunological responses relative to high-altitude exposure in mountaineers. Med. Sci. Sports Exerc..

[16] Karl J.P., Berryman C.E., Young A.J., Radcliffe P.N., Branck T.A., Pantoja-Feliciano I.G., Rood J.C., Pasiakos S.M. (2018). Associations between the gut microbiota and host responses to high altitude. Am. J. Physiol. Gastrointest. Liver Physiol..

[17] Wang J., Liu S., Xie Y., Xu C. (2023). Association analysis of gut microbiota-metabolites-neuroendocrine changes in male rats acute exposure to simulated altitude of 5500 m. Sci. Rep..

[18] Hidalgo-Cantabrana C., Delgado S., Ruiz L., Ruas-Madiedo P., Sánchez B., Margolles A. (2017). Bifidobacteria and Their Health-Promoting Effects. Microbiol. Spectr..

[19] Un-Nisa A., Khan A., Zakria M., Siraj S., Ullah S., Tipu M.K., Ikram M., Kim M.O. (2022). Updates on the Role of Probiotics Against Different Health Issues: Focus on *Lactobacillus*. Int. J. Mol. Sci..

[20] Zeng B., Zhang S., Xu H., Kong F., Yu X., Wang P., Yang M., Li D., Zhang M., Ni Q. (2020). Gut microbiota of Tibetans and Tibetan pigs varies between high and low altitude environments. Microbiol. Res..

[21] Amiri P., Hosseini S.A., Ghaffari S., Tutunchi H., Ghaffari S., Mosharkesh E., Asghari S., Roshanravan N. (2022). Role of Butyrate, a Gut Microbiota Derived Metabolite, in Cardiovascular Diseases: A comprehensive narrative review. Front. Pharmacol..

[22] Sun X., Li W., Chen G., Hu G., Jia J. (2025). *Faecalibacterium duncaniae* Mitigates Intestinal Barrier Damage in Mice Induced by High-Altitude Exposure by Increasing Levels of 2-Ketoglutaric Acid. Nutrients.

[23] Karl J.P., Fagnant H.S., Radcliffe P.N., Wilson M., Karis A.J., Sayers B., Wijeyesekera A., Gibson G.R., Lieberman H.R., Giles G.E. (2025). Gut microbiota-targeted dietary supplementation with fermentable fibers and polyphenols prevents hypobaric hypoxia-induced increases in intestinal permeability. Am. J. Physiol. Regul. Integr. Comp. Physiol..

[24] Weil J.V., Byrne-Quinn E., Sodal I.E., Friesen W.O., Underhill B., Filley G.F., Grover R.F. (1970). Hypoxic ventilatory drive in normal man. J. Clin. Investig..

[25] Bärtsch P., Maggiorini M., Ritter M., Noti C., Vock P., Oelz O. (1991). Prevention of high-altitude pulmonary edema by nifedipine. N. Engl. J. Med..

[26] Parati G., Agostoni P., Basnyat B., Bilo G., Brugger H., Coca A., Festi L., Giardini G., Lironcurti A., Luks A.M. (2018). Clinical recommendations for high altitude exposure of individuals with pre-existing cardiovascular conditions: A joint statement by the European Society of Cardiology, the Council on Hypertension of the European Society of Cardiology, the European Society of Hypertension, the International Society of Mountain Medicine, the Italian Society of Hypertension and the Italian Society of Mountain Medicine. Eur. Heart J..

[27] Lopez I., Aravena R., Soza D., Morales A., Riquelme S., Calderon-Jofré R., Moraga F.A. (2021). Comparison Between Pressure Swing Adsorption and Liquid Oxygen Enrichment Techniques in the Atacama Large Millimeter/Submillimeter Array Facility at the Chajnantor Plateau (5050 m). Front. Physiol..

[28] West J.B. (2002). Commuting to high altitude: Value of oxygen enrichment of room air. High. Alt. Med. Biol..

[29] Matamala Pizarro J., Aguayo Fuenzalida F. (2021). Mental health in mine workers: A literature review. Ind. Health.

[30] Mallet R.T., Burtscher J., Richalet J.P., Millet G.P., Burtscher M. (2021). Impact of High Altitude on Cardiovascular Health: Current Perspectives. Vasc. Health Risk Manag..

[31] Kayser B., Verges S. (2021). Hypoxia, energy balance, and obesity: An update. Obes. Rev..

[32] Chen S.M., Lin H.Y., Kuo C.H. (2013). Altitude training improves glycemic control. Chin. J. Physiol..

[33] Salazar G., Leyton B., Aguirre C., Anziani A., Weisstaub G., Corvalán C. (2021). Anthropometric and bioimpedance equations for fat and fat-free mass in Chilean children 7-9 years of age. Br. J. Nutr..

[34] Llames L., Baldomero V., Iglesias M.L., Rodota L.P. (2013). Valores del ángulo de fase por bioimpedancia eléctrica; estado nutricional y valor pronóstico [Values of the phase angle by bioelectrical impedance; nutritional status and prognostic value]. Nutr. Hosp..

[35] da Silva V.S., Vieira M.F.S. (2020). International Society for the Advancement of Kinanthropometry (ISAK) Global: International accreditation scheme of the competent anthropometrist. Rev. Bras. Cineantropometria Desempenho Humano.

[36] Evans M., Nguo K., Boneh A., Truby H. (2018). The Validity of Bioelectrical Impedance Analysis to Measure Body Composition in Phenylketonuria. JIMD Rep..

[37] Mottawea W., Sultan S., Landau K., Bordenave N., Hammami R. (2020). Evaluation of the Prebiotic Potential of a Commercial Synbiotic Food Ingredient on Gut Microbiota in an Ex Vivo Model of the Human Colon. Nutrients.

[38] Arias-Palencia N.M., Solera-Martínez M., Gracia-Marco L., Silva P., Martínez-Vizcaíno V., Cañete-García-Prieto J., Sánchez-López M. (2015). Levels and Patterns of Objectively Assessed Physical Activity and Compliance with Different Public Health Guidelines in University Students. PLoS ONE.

[39] Czarkowski M. (2014). Kolejna nowelizacja Deklaracj Helsińskiej [Helsinki Declaration–next version]. Pol. Merkur. Lekarski..

[40] Ohashi Y., Joki N., Yamazaki K., Kawamura T., Tai R., Oguchi H., Yuasa R., Sakai K. (2018). Changes in the fluid volume balance between intra- and extracellular water in a sample of Japanese adults aged 15–88 yr old: A cross-sectional study. Am. J. Physiol. Ren. Physiol..

[41] Martins P.C., Alves Junior C.A.S., Silva A.M., Silva D.A.S. (2023). Phase angle and body composition: A scoping review. Clin. Nutr. ESPEN.

[42] Torres-Mejías J., Rivarola E., Diaz E., Salazar G., Toro D., Villegas F., Gómez R., Cossio M., López M.A., Merellano-Navarro E. (2018). Somatotype and body composition of porters and guides on Mount Aconcagua, Argentina. Rev. Fac. Cien Med. Univ. Nac. Córdoba.

[43] McArdle B., Katch F., Katch V. (2015). Physical activity at medium and high altitudes. Fisiología del Ejercicio, Nutrición, Rendimiento y Salud.

[44] Brunser T.O. (2013). The role of bifidobacteria in the functioning of the human body. Rev. Chil. Nutr..

[45] Su Q., Zhuang D.H., Li Y.C., Chen Y., Wang X.Y., Ge M.X., Xue T.Y., Zhang Q.Y., Liu X.Y., Yin F.Q. (2024). Gut microbiota contributes to high-altitude hypoxia acclimatization of human populations. Genome Biol..

